# Long non-coding RNA-non-coding RNA activated by DNA damage inhibition suppresses hepatic stellate cell activation via microRNA-495-3p/sphingosine 1-phosphate receptor 3 axis

**DOI:** 10.1080/21655979.2022.2037841

**Published:** 2022-02-24

**Authors:** Lei Zou, Cuifen Shi, Dawei Wang, Juan Cheng, Qi Wang, Lei Wang, Guoya Yang

**Affiliations:** a Department of Infectious Diseases, Yancheng Second People’s Hospital, Yancheng, China; b Department of Gastroenterology, Yancheng Second People’s Hospital, Yancheng, China

**Keywords:** lncRNA NORAD, miR-495-3p, S1PR3, hepatic stellate cells, hepatic fibrosis

## Abstract

Hepatic fibrosis is a damage repair response caused by multiple factors. A growing body of research suggests that long non-coding RNAs (lncRNAs) are involved in a wide range of biological processes, and thus regulate disease progression, including hepatic fibrosis. In this study, we investigated the mechanisms of the long non-coding RNA-non-coding RNA activated by DNA damage (NORAD) in modulating hepatic fibrosis development. Platelet-derived growth factor-BB (PDGF-BB) was used to activate LX-2 hepatic stellate cells (HSCs). The expression of NORAD and microRNA (miR)-495-3p was determined by quantitative real-time polymerase chain reaction (qRT-PCR) analysis. The effects of PDGF-BB on LX-2 cell viability, migration, invasion, and apoptosis were evaluated using MTT (3-(4,5-dimethylthiazol-2-yl)-2,5-diphenyltetrazolium bromide), Transwell, flow cytometry, and Western blot assays. The activation of HSCs was further verified by examining the expression of the typical markers, alpha smooth muscle actin (α-SMA) and collagen I (Col1α1), using qRT-PCR and Western blot assays. StarBase and dual-luciferase reporter assays were used to assess the binding relationship between miR-495-3p and NORAD. The NORAD levels remarkably increased, whereas the miR-495-3p levels decreased, in PDGF-BB-treated LX-2 cells. miR-495-3p was a putative downstream target of NORAD. NORAD silencing played an anti-fibrotic role by targeting miR-495-3p; this was accomplished by hindering PDGF-BB-treated LX-2 cell viability, migration, and invasion, decreasing the levels of α-SMA and Col1α1, and promoting apoptosis. miR-495-3p protected against hepatic fibrosis by inhibiting sphingosine 1-phosphate receptor 3 (S1PR3) expression. In summary, NORAD silencing inhibited hepatic fibrosis by suppressing HSC activation via the miR-495-3p/S1PR3 axis.

## Graphical abstract

**Figure:**
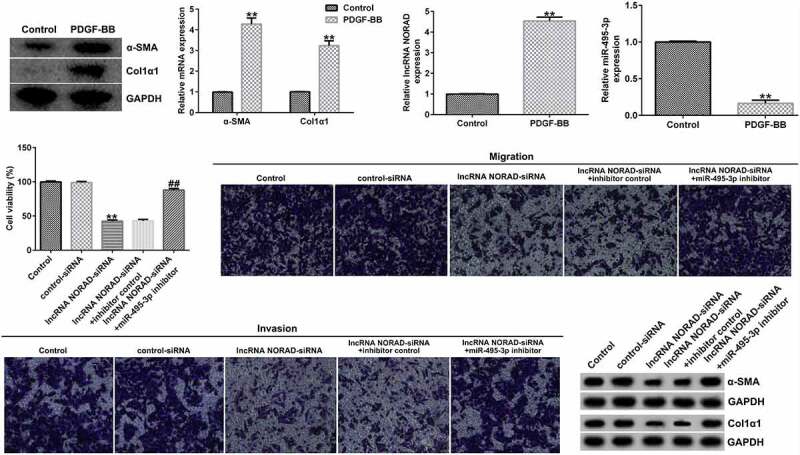

## Introduction

Hepatic fibrosis is the abnormal proliferation of connective tissue in the liver, resulting in an imbalance between the production and degradation of extracellular matrix (ECM), followed by the development of cirrhosis and liver failure, which leads to patient death [1,2]. In normal liver tissue, resting hepatic stellate cells (HSCs) are located between hepatocytes and hepatic sinusoidal endothelial cells [3]. HSCs can be activated by various pathogenic factors of chronic liver injury, such as persistent hepatitis virus infection, alcoholic fatty liver, nonalcoholic fatty liver, cholestasis, and autoimmune system diseases [4–7]. When such factors stimulate HSCs, quiescent HSCs become activated (fibroblasts), and start secreting large amounts of ECM, as well as pro-inflammatory factors, metalloproteinases, and tissue inhibitors of metalloproteinases, which trigger the development of fibrosis [8,9]. Therefore, the activation of HSCs is crucial in the development and progression of hepatic fibrosis.

In the fibrotic liver, HSCs undergo an activation process that manifests as contractility, proliferation, loss of vitamin A stores, and pathological production and deposition of ECM, including secretion of collagen I (Col1α1) and substantial production of alpha smooth muscle actin (α-SMA) [10,11]. Considering the critical role of HSCs in the development of hepatic fibrosis, inhibition of HSC activation is a major therapeutic target for the treatment of this condition. Platelet-derived growth factor (PDGF) is a basic protein stored in platelet alpha particles [12]. It stimulates a variety of cells, including fibroblasts, glial cells, and smooth muscle cells, which are stalled in the G0/G1 phase to enter the division and proliferation cycle [13]. Consistent with previous studies [14,15], we used PDGF-BB-induced LX-2 cells to mimic the activation of HSCs.

Long non-coding RNAs (lncRNAs) have transcripts longer than 200 bases but do not encode proteins [16]. They mediate diverse physiological and pathological processes such as inflammation and fibrosis by regulating gene expression at the epigenetic, transcriptional, and post-transcriptional levels [17–19]. In addition, a variety of lncRNAs have been implicated in the initiation and development of hepatic fibrosis. For instance, the lncRNA Lfar1 could promote HSC activation, hepatocyte apoptosis, and macrophage pyroptosis [20]. Shen et al. [21] revealed that knockdown of the lncRNA HULC could alleviate hepatic fibrosis by improving liver lipid deposition and hepatocyte apoptosis through the MAPK signaling pathway. The lncRNA NORAD, also known as LINC00657, is localized to chromosome 20: 36,045,622–36,050,960, and has a length of 5339 bp, including one exon [22]. Recently, a number of published papers suggested that NORAD was aberrantly expressed in some tumors, such as renal cancer [23], gastric cancer [24], ovarian cancer [25] and lung cancer [26]. The regulatory role of NORAD in liver- and fibrosis-related diseases has been widely reported. For instance, NORAD levels were found to be prominently increased in hepatocellular carcinoma (HCC) tissues and cells, serving as an oncogenetic factor in HCC [27–29]. Sur et al. [30] reported that NORAD silencing could suppress hepatocyte growth following hepatitis C virus infection. Moreover, in diabetic nephropathy, NORAD knockdown has been reported to inhibit HG-induced increases in the levels of inflammatory and fibrotic factors in human mesangial cells [31]. Furthermore, NORAD silencing lessened the fibrosis and inflammation response in diabetic cardiomyopathy [32]. A study by Xiong et al. [33] unveiled that NORAD accelerated fibrosis and apoptosis in hypoxia-exposed H9c2 cells, thus aggravating acute myocardial infarction. Such evidence suggests that NORAD plays an essential role in fibrosis. However, the role and mechanisms of the lncRNA NORAD in hepatic fibrosis remain unclear.

miRNAs are a class of endogenous single-stranded non-coding small RNAs that are 19–24 nucleotides in length [34]. They are involved in a series of critical cellular processes, including cell proliferation, regulation, differentiation, and metabolism [35]. Recent studies have shown that many miRNAs mediate the activation, proliferation, and regulation of HSCs by regulating their target genes involved in signaling pathways such as the transforming growth factor-beta (TGF-β)/Smad [36], Wnt/beta-catenin [37], phosphatidylinositol 3-kinase (PI3K)/Protein kinase B (AKT) [38], and NF-kappaB (NF-κB) pathways [39], and thus play a pivotal role in the development and progression of hepatic fibrosis. It has been shown that miR-495-3p plays a protective role in pulmonary fibrosis by downregulating S1PR3 expression [40]. S1PR3, a critical factor in fibrosis, is significantly overexpressed in hepatic fibrosis; hence, the silencing of its gene inhibits hepatic fibrosis [41,42]. However, the role of miR-495-3p in hepatic fibrosis is unclear.

Using bioinformatics tools, we found a direct binding site between NORAD and miR-495-3p. Therefore, in this study, we hypothesized that NORAD might play an important role in hepatic fibrosis by regulating the miR-495-3p/S1PR3 axis. Therefore, this study was aimed at investigating the role of the NORAD/miR-495-3p-S1PR3 axis in the activation of HSCs, and revealing its mechanism, thereby providing a new theoretical basis for the treatment of hepatic fibrosis.

## Materials and methods

### Cell culture and HSC activation

Human HSCs (LX-2 cells) were purchased from American Type Culture Collection (USA) and cultured in Dulbecco’s modified easy medium (DMEM) with 10% fetal bovine serum (FBS) and 1% penicillin-streptomycin. For HSC activation, LX-2 cells were serologically starved in FBS-free DMEM for 24 h and then treated with 10 ng/mL PDGF-BB (ACROBiosystems, Beijing, China) [14,15,43].

### Cell transfection

miR-495-3p inhibitor, inhibitor control, miR-495-3p mimic, mimic control, control siRNA (5′-GCGCGATAGCGCGAATATA-3′), lncRNA NORAD siRNA (5′-AATAGAATGAAGACCAACCGC-3′), control plasmid, and S1PR3 plasmid were designed and synthesized by Ribobio (Guangzhou, China). These oligonucleotides were transfected into LX-2 cells using Lipofectamine 2000 reagent (Thermo Fisher Scientific, Inc., Shanghai, China). The transfected cells were cultured at 37°C under 5% CO_2_ for 48 h.

### qRT-PCR analysis

Total RNA was extracted from transfected LX-2 cells using TRIzol reagent. cDNA was synthesized according to the instructions of a reverse transcription kit (Thermo Fisher Scientific), and PCR was performed using the cDNA as the template. The amplification conditions were as follows: 95°C for 2 min; 95°C for 15s, 60°C for 30s, and 70°C for 1 min, for a total of 40 cycles. Three replicate experiments were set up. The CT value of each well was recorded, and the average of the three replicate wells was used as the final result. U6 and GAPDH were used as internal references and the 2^−ΔΔCT^ method [44] was used for analysis. Primer sequences for PCR were listed as following:

GAPDH forward, 5’-CTTTGGTATCGTGGAAGGACTC-3’; reverse, 5’-GTAGAGGCAGGGATGATGTTCT-3’; U6 forward, 5’-GCTTCGGCAGCACATATACTAAAAT-3’; reverse, 5’-CGCTTCACGAATTTGCGTGTCAT-3’; lncRNA NORAD forward, 5’-TGATAGGATACATCTTGGACATGGA-3’; rever-se, 5’-AACCTAATGAACAAGTCCTGACATACA-3’; miR-495-3p forward, 5ʹ-ACACTCCAGCTGGGAAACAAACATGGTGCA-3ʹ; reverse, 5ʹ-TGGTGTCGTGGAGTCG-3ʹ; S1PR3 forward, 5ʹ-GTGATCCTCTACGCACGCATC-3’; reverse, 5ʹ-CGCTCCGAGTTGTTGTGGT-3’; α-SMA forward, 5’-GTGCTGTCCCTCTATGCCTCTGG-3’; reverse, 5’-GGCACGTTGTGAGTCACACCATC-3’; Col1α1 forward, 5’-CCTGCCTGCTTCGTGTAAA-3’; reverse, 5’-TTGAGTTTGGGTTGTTGGTCT-3’.

### MTT assay [45]

After 24 h from transfection, each group of cells was digested with trypsin, and the cell suspension was adjusted to 3 × 10^3^ cells/well and inoculated in 96-well plates at 37°C under 5% CO_2_. After removing the medium from the wells, 110 µL of dimethyl sulfoxide (DMSO) was added to each well and then shaken at low speed for 10 min to dissolve the crystals. Finally, the cell growth curve was plotted by measuring the optical density (OD) value at 492 nm for each well using an enzyme marker, with time as the horizontal coordinate and the absorbance value as the vertical coordinate. The experiment was repeated thrice.

### Transwell assay [46]

A Transwell chamber pre-coated with or without Matrigel was used for measuring cell invasion and migration. Briefly, 1 × 10^5^ cells/well LX-2 cells were inoculated in 24-well plates. The LX-2 cells were digested with trypsin and then re-suspended in DMEM containing 1% FBS to make a cell suspension. The cell suspension was added to the upper chamber of the 24-well plate at 100 μL/well, and the lower chamber was filled with 600 μL of DMEM containing 10% FBS. The Transwell chamber was then incubated for 24 h at 37°C under 5% CO_2_ and then fixed with 4% paraformaldehyde. The cells were stained with 0.1% crystal violet for 5 min at room temperature, and the non-migrating cells were removed with a cotton swab. Three fields were randomly selected for observation under an immunofluorescence microscope.

### Flow cytometry analysis [47]

Logarithmic-growth-phase LX-2 cells were selected and inoculated into 6-well plates at a density of 4 × 10^5^ cells/well. After 48 h from transfection, 195 µL of Annexin V-FITC binding buffer (Beyotime, Shanghai, China) was added to each group, and the cells were gently re-suspended. Then, 5 µL Annexin V-FITC (Beyotime, Shanghai, China) and 10 µL propidium iodide (Beyotime, Shanghai, China) were added, and the cells were incubated for 20 min at room temperature while protected from light. The apoptosis rate was analyzed using flow cytometry.

### Western blot assay [48]

LX-2 cells were collected after transfection and fully lysed using lysis solution to obtain total protein. After gel electrophoresis and nitrocellulose membrane blotting, the membranes were blocked with TBST containing 5% skim milk powder for 2 h. Thereafter, the membranes were transferred to dilutions containing GAPDH antibody (ab9485, 1:1000, Abcam, Shanghai, China), α-SMA antibody (ab5694, 1:1000, Abcam), Col1α1 antibody (ab166606, 1:1000, Abcam), and cleaved-Caspase3 antibody (ab32042, 1:1000, Abcam) and incubated at 4°C overnight. The membranes were washed thrice with TBST (15 min each) and incubated with the secondary antibody (ab7090, 1:1000, Abcam) at room temperature for 2 h. Finally, the membranes were developed using ECL chemiluminescent reagent.

### Dual-luciferase reporter assay [49]

The wild type (lncRNA NORAD-WT) and mutant (lncRNA NORAD-MUT) 3′-untranslated regions (UTRs) of NORAD were cloned into the pGL3 vector (Promega) to construct a pGL3-lncRNA NORAD-WT vector and pGL3-lncRNA NORAD-Mutant vector. Then, 0.4 μg pGL3-NORAD-WT or pGL3-NORAD-Mutant and 50 mM miR-495-3p mimic or mimic control were co-transfected into LX-2 cells. The luciferase activity was measured using a luciferase reporter assay kit, in accordance with the instructions. Finally, the luciferase activity was normalized to *Renilla* activity.

## Statistical analysis

SPSS 17.0 software was used for statistical analysis of data. The Student’s t-test was used for comparison between 2 groups, and one-way ANOVA followed by Tukey’s test was used for analysis among three or more groups. P < 0.05 indicated that the difference was statistically significant.

## Results

### NORAD had a competitive relationship with miR-495-3p

**Figure 1.**
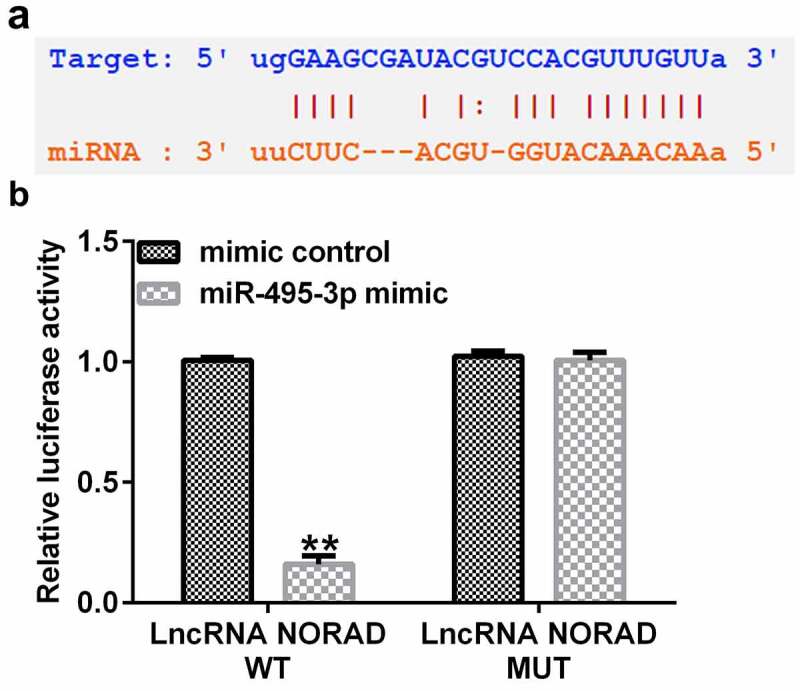
LncRNA NORAD was the complementary endogenous RNA (ceRNA) for miR-495-3p. (a) Putative complimentary sequences between NORAD and miR-495-3p were predicted by StarBase. (b) Dual-luciferase reporter assay confirmed the binding relationship between NORAD and miR-495-3p. Data were expressed as means ± SD, and experiments were repeated for at least for 3 times. **p < 0.01 vs. mimic control group.

To predict the putative relationship between NORAD and miR-495-3p, StarBase was used. As shown in Figure 1a, NORAD had complementary sequences for miR-495-3p. Furthermore, in the dual-luciferase reporter assay, the luciferase activity signal was weaker in the pGL3-NORAD-WT and miR-495-3p mimic-transfected group than in the pGL3-NORAD-WT and mimic control co-transfection group (Figure 1b). Moreover, there were no apparent differences in the pGL3-NORAD-Mutant transfection groups. These results illustrated that NORAD was the complimentary endogenous RNA (ceRNA) for miR-495-3p.

### NORAD was promoted, whereas miR-495-3p was inhibited, in PDGF-BB-induced LX-2 cells

**Figure 2.**
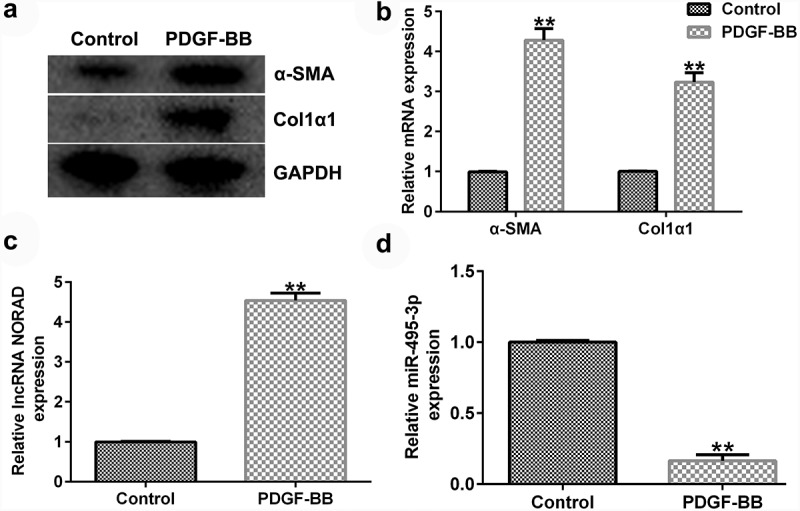
LncRNA NORAD levels were elevated, whereas miR-495-3p levels were lowered, in activated HSCs. LX-2 cells were treated with 10 ng/mL PDGF-BB for 24 h to activate HSCs. (a) Western blot assay verified high expression levels of α-SMA and Col1α1 in PDGF-BB-induced LX-2 cells. (b) qRT-PCR verified the high mRNA expression levels of α-SMA and Col1α1 in in PDGF-BB-induced LX-2 cells. (c) qRT-PCR verified high expression levels of NORAD in PDGF-BB-induced LX-2 cells. (d) qRT-PCR verified poor expression of miR-495-3p in PDGF-BB-induced LX-2 cells. Data were expressed as means ± SD, and experiments were repeated for at least for 3 times. **p < 0.01 vs. Control group.

To study the expression of NORAD and miR-495-3p in activated HSCs, LX-2 cells were treated with 10 ng/mL PDGF-BB for 24 h to activate HSCs, as described before [14,15]. We first detected the expression of α-SMA and Col1α1, PDGF-BB activated fibrosis markers, in PDGF-BB treated LX-2 cells, and the data indicated that compared with the control group, the protein and mRNA expression of α-SMA and Col1α1, PDGF-BB activated fibrosis markers, in PDGF-BB treated LX-2 cells significantly enhanced (Figure 2a and b). Then, qRT-PCR analysis was conducted to examine the effects of HSC activation on NORAD and miR-495-3p expression. As shown in Figures 2c and 2d, NORAD expression was noticeably elevated, whereas miR-495-3p expression was hindered, after PDGF-BB treatment. These results suggested that NORAD and miR-495-3p might modulate the development of hepatic fibrosis.

### NORAD silencing protected against hepatic fibrosis by upregulating miR-495-3p levels

**Figure 3.**
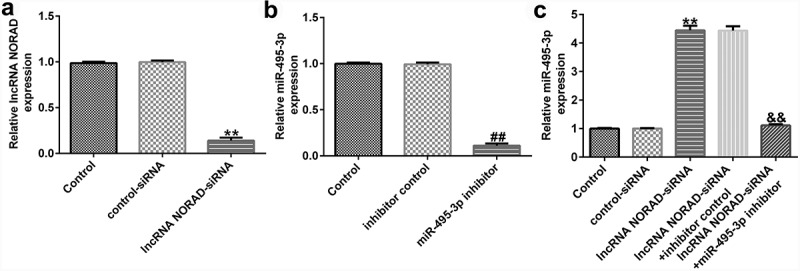
Transfection efficacy of lncRNA NORAD and miR-495-3p in PDGF-BB-triggered LX-2 cells. (a) PDGF-BB-treated LX-2 cells were transfected with control siRNA and lncRNA NORAD siRNA. NORAD expression was determined by qRT-PCR analysis. (b) PDGF-BB-treated LX-2 cells were transfected with inhibitor control and miR-495-3p inhibitor. miR-495-3p expression was determined by qRT-PCR analysis. (c) PDGF-BB-treated LX-2 cells were transfected and co-transfected with control siRNA, lncRNA NORAD siRNA, and miR-495-3p inhibitor. miR-495-3p expression was determined by qRT-PCR analysis. Data were expressed as means ± SD, and experiments were repeated for at least for 3 times. **p < 0.01 vs. control-siRNA; ##p < 0.01 vs. inhibitor control; &&p < 0.01 vs. lncRNA NORAD-siRNA+inhibitor control.

To confirm the regulatory role of the NORAD/miR-495-3p axis in hepatic fibrosis, LX-2 cells were pre-treated with PDGF-BB for 24 h and transfected with lncRNA NORAD siRNA and/or miR-495-3p inhibitor. As depicted in Figure 3a, after transfection with lncRNA NORAD siRNA, the expression of NORAD prominently decreased, suggesting that the transfection was successful. Moreover, miR-495-3p was successfully knocked down upon transfection with miR-495-3p inhibitor (Figure 3b). Furthermore, we found that lncRNA NORAD siRNA could enhance miR-495-3p expression; this enhancement could be partially reversed by co-transfection with miR-495-3p inhibitor (Figure 3c).

**Figure 4.**
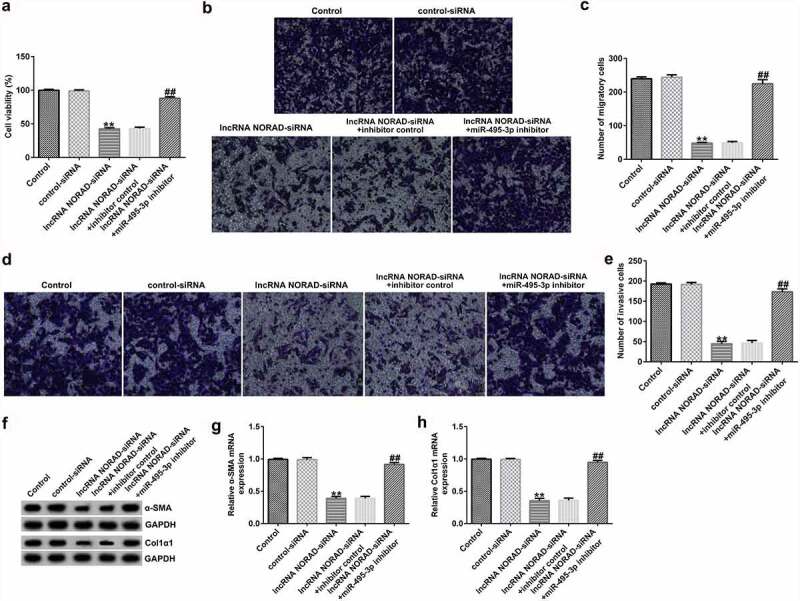
Knockdown of lncRNA NORAD inhibited cell viability, migration, and invasion in activated HSCs by targeting miR-495-3p. PDGF-BB-treated LX-2 cells were transfected with control siRNA, lncRNA NORAD siRNA, lncRNA NORAD siRNA + inhibitor control, or lncRNA NORAD siRNA + miR-495-3p inhibitor for 24 h. (a) MTT assay was conducted to measure cell viability. (b, c) Transwell assay was performed to evaluate cell migration (magnification: 200×). (d, e) Transwell assay was performed to evaluate cell invasion (magnification: 200×). (f) Markers of activated HSCs (α-SMA and Col1α1) were detected by Western blot assay. (g) mRNA level of α-SMA was verified by qRT-PCR analysis. (h) mRNA level of Col1α1 was verified by qRT-PCR analysis. Data were expressed as means ± SD, and experiments were repeated for at least for 3 times. **p < 0.01 vs. control-siRNA; ##p < 0.01 vs. lncRNA NORAD-siRNA+inhibitor control.

**Figure 5.**
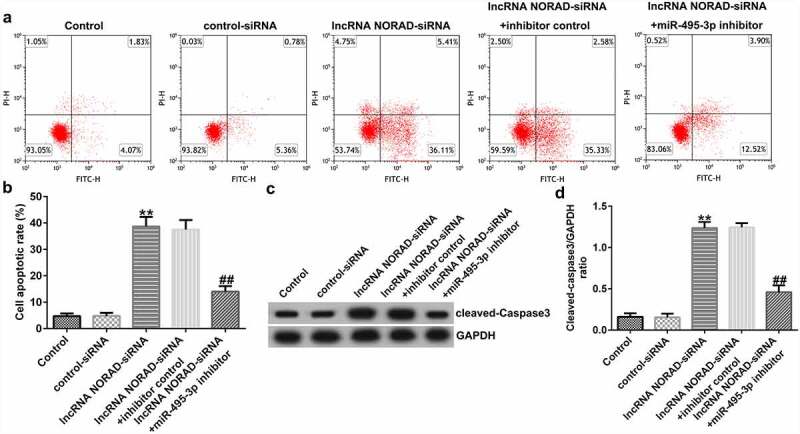
Knockdown of lncRNA NORAD enhanced cell apoptosis in activated HSCs by targeting. PDGF-BB-treated LX-2 cells were transfected with control-siRNA, lncRNA NORAD siRNA, lncRNA NORAD siRNA + inhibitor control, or lncRNA NORAD siRNA + miR-495-3p inhibitor for 24 h. (a, b) Cell apoptosis was examined by flow cytometry analysis. (c) Western blot assay was used to determine cleaved-Caspase3 levels. (d) Cleaved-Caspase3/GAPDH ratio was quantified in accordance with the results shown in panel C. Data were expressed as means ± SD, and experiments were repeated for at least for 3 times. **p < 0.01 vs. control-siRNA; ##p < 0.01 vs. lncRNA NORAD-siRNA+inhibitor control.

The functional experiment further illustrated the role of the NORAD/miR-495-3p axis in hepatic fibrosis. As demonstrated in Figure 4a-4e, the MTT and Transwell results revealed that NORAD silencing could inhibit PDGF-BB-induced LX-2 cell viability, migration, and invasion. However, co-transfection with miR-495-3p inhibitor reversed the inhibitory effects induced by lncRNA NORAD siRNA on PDGF-BB-treated LX-2 cells. Previous studies have shown that α-SMA and Col1α1 are typical markers for evaluating HSC activation. The Western blot and qRT-PCR analysis results in Figure 4f-4h indicate that NORAD silencing decreased α-SMA and Col1α1 expression compared with that in the control siRNA group. Moreover, co-transfection with miR-495-3p inhibitor could partially reverse the depletion of α-SMA and Col1α1 triggered by lncRNA NORAD siRNA (Figure 4f-4h). In addition, flow cytometry analysis indicated that NORAD knockdown promoted the apoptosis of PDGF-BB-treated LX-2 cells (Figure 5a and 5b), and increased cleaved-Caspase3 levels (Figure 5c and 5d). Nevertheless, the enhancement in cell apoptosis and cleaved-Caspase3 levels triggered by lncRNA NORAD siRNA could be counteracted by miR-495-3p inhibitor to some degree (Figure 5a-5d).

### miR-495-3p played an anti-fibrotic role by targeting S1PR3

**Figure 6.**
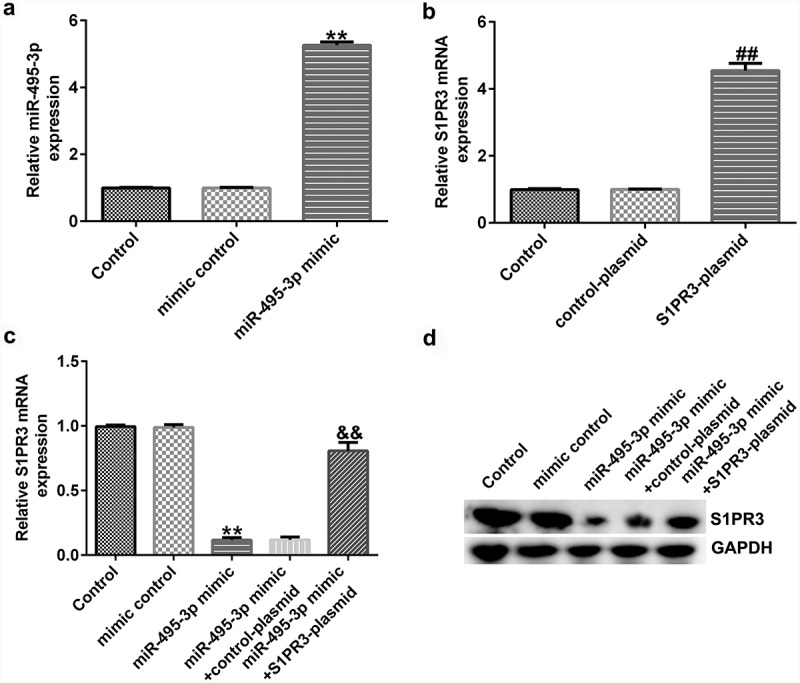
miR-495-3p negatively modulated S1PR3 expression in PDGF-BB-treated LX-2 cells. PDGF-BB-treated LX-2 cells were transfected with mimic control, miR-495-3p mimic, control plasmid, S1PR3 plasmid, miR-495-3p mimic + control plasmid, or miR-495-3p mimic + S1PR3 plasmid for 24 h. (a) Transfection efficiency of miR-495-3p was determined by qRT-PCR analysis. (b) Transfection efficiency of S1PR3 was determined by qRT-PCR analysis. (c) mRNA expression of S1PR3 after co-transfection with miR-495-3p mimic and S1PR3 plasmid was detected by qRT-PCR assay. (d) Protein expression of S1PR3 after co-transfection with miR-495-3p mimic and S1PR3 plasmid was detected by Western blot assay. Data were expressed as means ± SD, and experiments were repeated for at least for 3 times. **p < 0.01 vs. mimic control; ##p < 0.01 vs. control-plasmid; &&p < 0.01 vs. miR-495-3p mimic+control-plasmid.

We finally investigated the role and mechanism of miR-495-3p in hepatic fibrosis. A previous study illustrated the binding relationship between miR-495-3p and S1PR3 [40] in pulmonary fibrosis. Hence, we performed functional experiments to unveil the miR-495-3p/S1PR3 regulatory axis in hepatic fibrosis. Firstly, the transfection efficacy results in Figure 6a and 6b implied that miR-495-3p mimic transfection could increase miR-495-3p expression, whereas S1PR3 plasmid transfection could elevate S1PR3 levels in PDGF-BB-treated LX-2 cells. Subsequently, the co-transfection assay showed that, compared with that in the mimic control group, S1PR3 mRNA and protein expression was restrained in the miR-495-3p mimic group (Figure 6c and 6d). Moreover, the restraint in S1PR3 expression induced by miR-495-3p mimic could be counterbalanced by co-transfection with S1PR3 plasmids (Figure 6c and 6d). These data demonstrated that miR-495-3p could negatively regulate S1PR3 expression.

**Figure 7.**
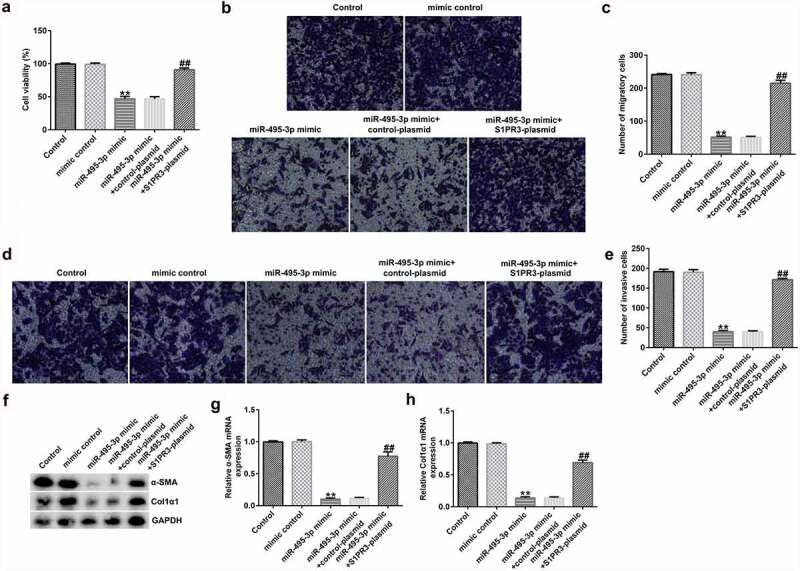
miR-495-3p restrained PDGF-BB-induced enhancement in LX-2 cell proliferation, migration, and invasion by inhibiting S1PR3 levels. PDGF-BB-treated LX-2 cells were transfected with mimic control, miR-495-3p mimic, control plasmid, S1PR3 plasmid, miR-495-3p mimic + control plasmid, or miR-495-3p mimic + S1PR3 plasmid for 24 h. (a) MTT assay was used to evaluate cell viability. (b, c) Migratory abilities were determined by Transwell assay (magnification: 200×). (d, e) Invasive capabilities were verified by Transwell assay (magnification: 200×). (f) Western blot assay was used to detect α-SMA and Col1α1 protein expression. (g) mRNA expression of α-SMA was examined by qRT-PCR analysis. (h) mRNA expression of Col1α1 was examined by qRT-PCR analysis. Data were expressed as means ± SD, and experiments were repeated for at least for 3 times. **p < 0.01 vs. mimic control; ##p < 0.01 vs. miR-495-3p mimic+control-plasmid.

**Figure 8.**
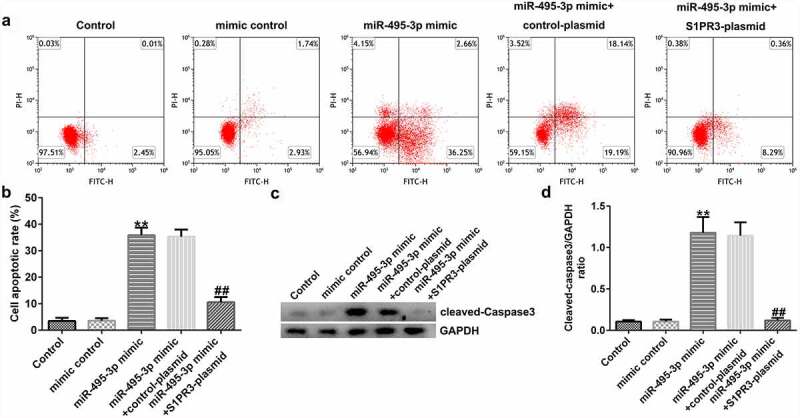
S1PR3 reversed the promotive effects of miR-495-3p mimic on PDGF-BB-treated LX-2 cell apoptosis. (a, b) Cell apoptosis was detected by flow cytometry analysis. (c) Protein expression of cleaved-Caspase3 was assessed by Western blot assay. (d) Cleaved-Caspase3/GAPDH ratio was quantified as depicted in panel C. Data were expressed as means ± SD, and experiments were repeated for at least for 3 times. **p < 0.01 vs. mimic control; ##p < 0.01 vs. miR-495-3p mimic+control-plasmid.

MTT, Transwell, qRT-PCR, Western blot, and flow cytometry analyses further elucidated the mechanisms of the miR-495-3p/S1PR3 axis in hepatic fibrosis regulation. As shown in Figure 7a-7h, compared with those in the mimic control group, cell viability, migration, invasion, and α-SMA and Col1α1 expression were evidently lower in miR-495-3p mimic-transfected LX-2 cells under PDGF-BB treatment. However, the reduction induced by miR-495-3p could be restored by transfection with S1PR3-overexpressing S1PR3 plasmids. On the contrary, miR-495-3p mimic could enhance LX-2 cell apoptosis as well as cleaved-Caspase3 levels after PDGF-BB treatment (Figure 8a-8d). Nevertheless, the enhancement in cell apoptosis induced by miR-495-3p mimic could be partially counterbalanced by S1PR3 plasmid transfection.

## Discussion

The lncRNA NORAD, also known as LINC00657, has been proved to be aberrantly expressed in various types of tumors [23–26]. Evidence has also suggests that NORAD plays an essential role in fibrosis [30–33]. In our study, after inducing LX-2 cells with PDGF-BB, we observed significantly increased NORAD expression. In addition, the functional experiments suggested that NORAD knockdown could counteract abnormal cell viability, migration, invasion, α-SMA and Col1α1 levels, and cell apoptosis induced by HSC activation. Collectively, our data indicate that NORAD exhibits anti-fibrogenic activity in hepatic fibrosis.

miR-495 is a non-coding RNA molecule located at the human chromosome 14q32.31 locus [50]. Numerous studies have shown that miR-495 is involved in immune processes. For instance, Yang et al. [51] demonstrated that miR-495-3p was downregulated in oral squamous cell carcinoma (OSCC) and that overexpression of miR-495-3p could inhibitor OSCC progression and immune evasion. Li et al. [52] illustrated that miR-495 could promote the senescence of mesenchymal stem cells, which was related to immune balance. Moreover, a study by Hu et al. [53] revealed that miR-495 modulates M1/M2 polarization and insulin resistance in type 2 diabetes. In sepsis, miR-495-3p downregulation could enhance sepsis-related inflammation [54]. In HCC, miR-495 functions as a tumor suppressor [55–57]. In the fibrosis realm, Guo et al. [58] discovered that miR-495 could suppress the growth of fibroblasts in hypertrophic scars. Wang et al. [59] verified that miR-495 could restrain cardiac fibroblasts by targeting NOD1. Hou et al. [60] found that miR-495 overexpression could alleviate bladder fibrosis in interstitial cystitis. Moreover, miR-495-3p was reported to target S1PR3 to mitigate pulmonary fibrosis [28]. In our study, we first found that miR-495-3p was a downstream target of NORAD. miR-495-3p downregulation could reverse the effects of lncRNA NORAD siRNA on HSC activation. Furthermore, the functional experiments revealed that miR-495-3p could protect against HSC activation by targeting S1PR3.

## Conclusion

NORAD inhibition could suppress HSC activation by modulating the miR-495-3p/S1PR3 axis, providing new insights for hepatic fibrosis treatment.

## Data Availability

The datasets used and/or analyzed during the current study are available from the corresponding author upon reasonable request.
